# Design, Synthesis and Evaluation of Antiproliferative Activity of New Benzimidazolehydrazones

**DOI:** 10.3390/molecules21050579

**Published:** 2016-04-30

**Authors:** Valentina Onnis, Monica Demurtas, Alessandro Deplano, Gianfranco Balboni, Anna Baldisserotto, Stefano Manfredini, Salvatore Pacifico, Sandra Liekens, Jan Balzarini

**Affiliations:** 1 Dipartimento di Scienze della Vita e dell’Ambiente, Sezione di Scienze Farmaceutiche, Farmacologiche e Nutraceutiche, Università degli Studi di Cagliari, Via Ospedale 72, Cagliari I-09124, Italy; monicademurtas@tiscali.it (M.D.); deplano.a@gmail.com (A.D.); gbalboni@unica.it (G.B.); 2 Dipartimento di Scienze della Vita e Biotecnologie, Università degli Studi di Ferrara, Via Fossato di Mortara 17-19, Ferrara I-44121, Italy; bldnna@unife.it (A.B.) mv9@unife.it (S.M.); 3 Dipartmento di Scienze Chimiche e Farmaceutiche, Università degli Studi di Ferrara, Via Fossato di Mortara 17-19, Ferrara I-44121, Italy; salvatore.pacifico@unife.it; 4 KU Leuven—University of Leuven, Department of Microbiology and Immunology, Rega Institute for Medical Research, Laboratory of Virology and Chemotherapy, Leuven B-3000, Belgium; sandra.liekens@rega.kuleuven.be (S.L.); jan.balzarini@rega.kuleuven.be (J.B.)

**Keywords:** benzimidazoles, hydrazones, antiproliferative activity

## Abstract

The synthesis and antiproliferative activity of new benzimidazole derivatives bearing an hydrazone mojety at the 2-position is described. The new *N*′-(4-arylidene)-1*H*-benzo[*d*]imidazole-2-carbohydrazides were evaluated for their cytostatic activity toward the murine leukemia (L1210), human T-cell leukemia (CEM), human cervix carcinoma (HeLa) and human pancreas carcinoma cells (Mia Paca-2). A preliminary structure-activity relationship could be defined. Some of the compounds possess encouraging and consistent antiproliferative activity, having IC_50_ values in the low micromolar range.

## 1. Introduction

As recently reviewed [1], benzimidazole is a privileged structure in medicinal chemistry because of its broad range of biological activities. The benzimidazole ring is present in some clinically used drugs, such as proton pump inhibitors, antihelmintic compounds, the antiviral enviroxime and the antihistaminic astemizole, but it may also display antimycobacterial, antimicrobial, anticonvulsant, analgesic, anti-inflammatory, anti-diabetic, antiprotozoal, antipsychotic, antioxidant and antitumoral properties [1].

Several series of benzimidazole derivatives have shown antiproliferative activity. Moreover, benzimidazole-5-carboxylic acid derivatives induced cell death in leukemic cells [2]. The antiproliferative activity of benzimidazole derivatives has been correlated to multi-target kinase inhibition [3,4]. Furthermore, benzimidazole carbamates showed antitubulin activity [5,6] and benzimidazole-carbazole conjugates were described to stabilize human telomeric DNA and to inhibit telomerase and topoisomerase I in cancer cells [7,8]. Benzimidazole has also been reported as a new scaffold endowed with sirtuin 1 and 2 inhibitory activity. These compounds also showed cytotoxicity against the breast cancer cell lines MCF-7 and MDA-MB-468 [9,10].

Hydrazone is another biologically active pharmacophore group. *N*-(1′-Naphthyl)-3,4,5-trimethoxybenzohydrazide has been reported as a potential anti-leukemia agent acting as a microtubule destabilizer [11]. 2-*N*-Heteroaryl caffeine hydrazones demonstrated high activity toward T-lymphoblastic leukemia cells and inhibited both RNA and DNA synthesis and mitosis [12]. 4-(2-Fluorophenoxy)quinolineacylhydrazones showed excellent antiproliferative activity and c-Met kinase inhibitory activity [13]. Salicylaldehyde isonicotinoylhydrazone analogs behaved as iron chelators especially in MCF-7 breast adenocarcinoma cells and this was correlated to their cytotoxic activity [14], while salicylaldehyde 1-arylmethyl-3-aryl-1*H*-pyrazole-5-carbohydrazide hydrazone derivatives inhibited the growth of A549 lung cancer cells [15]. We have previously reported on 2-arylamino-6-trifluoromethyl-3-(hydrazinocarbonyl)pyridines [16] showing *in vitro* inhibitory activity against human tumour cell lines at low micromolar to nanomolar concentrations. Furthermore 4-(diethylamino)salycilaldehyde hydrazones showed potent cytotoxicity against human tumour cell lines but showed no toxicity in athymic nude mice [17]. Although the biological activity of benzimidazole and hydrazine structures has been well documented, an extensive literature search revealed very few efforts to combine these two important moieties in a single molecular scaffold. Few studies described the anti-inflammatory and antimicrobial activity of benzimidazole-5-carboxylate and its hydrazone derivatives [18] and the antiproliferative activity of *N*-1-benzoimidazole acetohydrazides against various tumor cell lines [19]. Therefore, in this explorative work, based on the antiproliferative activity of benzimidazole and hydrazone containing structures we decided to investigate, in a dualistic approach [20], new benzimidazole-hydrazone compounds, namely *N*′-(4-arylidene)-1*H*-benzo[*d*]imidazole-2-carbohydrazides and investigated their antiproliferative activity.

## 2. Results

### 2.1. Chemistry

**Scheme 1:**
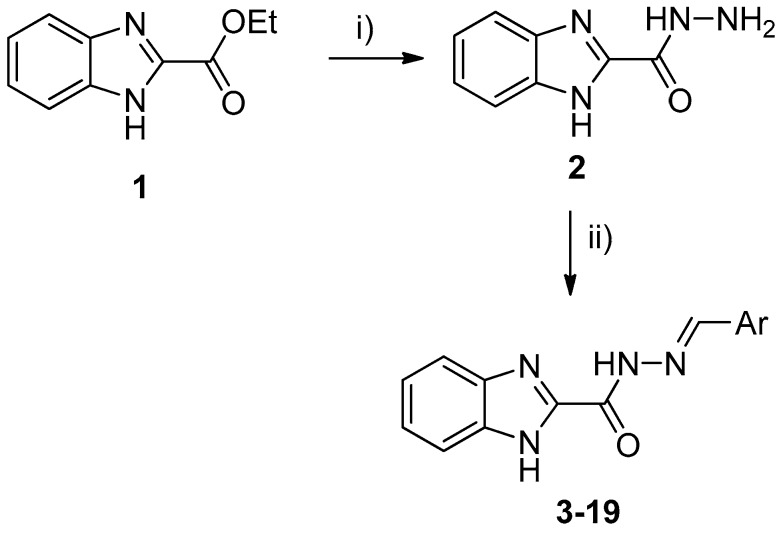
Synthesis of hydrazones **3**–**19**. *Reagents and conditions*: (i) NH_2_NH_2_·H_2_O, EtOH, reflux, 3 h; (ii) ArCHO, EtOH reflux, 5 h.

The target hydrazones **3**–**19** were synthesized as shown in Scheme 1. High yields of 1H-benzo[*d*]imidazole-2-carbohydrazide (**2**) were achieved upon refluxing for 3 h in an ethanolic solution of the corresponding ethyl ester **1** and hydrazine hydrate. Hydrazones **3**–**19** were obtained in good to excellent yield by coupling the hydrazide **2** with the appropriate hydroxyarylaldehydes in ethanol. All the newly synthesized compounds gave corrected analytical data. The IR and NMR spectral data are consistent with the assigned structure. According to the literature, the presence of a single downfield resonating (8.49–9.79 ppm) CH=N signal indicates the exclusive formation of *E*-isomers [21].

### 2.2. Antiproliferative Activity

The synthesized hydrazones **3**–**19** were evaluated *in vitro* for their inhibitory effects on the proliferation of murine leukemia (L1210), human T-lymphoblastic leukemia (CEM), human cervix carcinoma (HeLa) and human pancreas carcinoma (Mia Paca-2) cells. The presence of a 2-hydroxyl group on the arylidene moiety favourably modulates antiproliferative activity (Table 1).

## 3. Discussion

Hydrazones **10** and **16** inhibited the growth of all tested cell lines with low (<10 μM) micromolar IC_50_ values. The replacement of the 2-hydroxynaphthyl group of compound **16** with the 2-hydroxyphenyl resulted in the hydrazone **3** endowed with reduced antiproliferative activity against HeLa and Mia Paca-2 cells. The shift of the hydroxyl group from the 2-position to the 3- or 4-position led to poorly active compounds **4** and **5**. The introduction of a second 4-hydroxyl group (compound **6**) caused an increase in activity with respect to the 2-hydroxy analog **3**, whereas the presence of the 2,5-dihydroxybenzylidene group (compound **7**) led to a clear reduction in inhibitory activity as compared with compounds **4** and **6**. The introduction of a third hydroxyl group (compounds **8** and **9**) resulted in reduction or loss of cytostatic activity. A comparison of substituent effects revealed that the introduction of a 2-hydroxy-5-halobenzylidene moiety led to compounds **14** and **15** endowed with a better antiproliferative effect as compared with 2,5-dihydroxybenzylidene and 2-hydroxybenzylidene analogs **7** and **4**. The hydrazone of 5-chlorosalicylaldehyde **14** showed antiproliferative activity at single digit micromolar IC_50_ values on all tumor cell lines, while the 5-bromo derivative **15** was less active on Mia Paca-2 cells. The introduction of the 2-hydroxy-4-methoxybenzylidene moiety (compound **10**) caused an increase in activity as compared with 2,4-dihydroxybenzylidene and 2-hydroxybenzylidene analogs **6** and **3**. A reduction in activity was produced by the shift of the ether group from the 4- to the 3-position (hydrazone **11**), while the shift of the hydroxyl group from the 2- to the 3-position (compound **12**) led to a drop in activity. The replacement of the 4-methoxy group with a 4-(diethylamino) group was tolerated although hydrazone **13** was less active as compared with hydrazones **3** and **6**. The hydrazones **17**–**19** lacking hydroxyl groups are completely inactive.

The efficacy of any drug depends on its high oral bioavailability, so we assessed the potential bioavailability of the most active compounds, using the adsorption, distribution, metabolism and elimination (ADME) prediction method by molinspiration (http://www.molinspiration.com/cgi-bin/properties).

According to Lipinski’s rule of five [22] a compound to become a successful drug candidate, should have a molecular weight ≤500, a log *p* ≤ 5, hydrogen bond donor sites ≤5 and hydrogen bond acceptor sites (N and O atoms) ≤10. Predictions of the ADME properties for studied compounds (Table 2) showed that all the active compounds fulfilled this rule, similarly to clinically used drugs. Furthermore, the number of rotable bonds is important for conformational flexibility of the molecule. The total polar surface area (TPSA) is another key property that has been linked to drug bioavailability. These two parameters, *i.e.*, the number of rotable bonds in a molecule ≤10 and polar surface area ≤140 Å are essential for a good oral bioavailability [23]. Theoretically, all hydrazones **3**, **6**–**8**, **10**, **11**, **13**–**16** should present good passive oral absorption and differences in their bioactivity cannot be attributed to this property.

## 4. Materials and Methods

### 4.1. General Information

All commercially available solvents and reagents were used without further purification. ^1^H- and ^13^C-NMR spectra were recorded on an Inova 500 spectrometer (Varian, Palo Alto, CA, USA). The chemical shifts (δ) are reported in part per million downfield from tetramethylsilane (TMS), which was used as internal standard, and the spectra were recorded in hexadeuteriodimethylsulphoxide (DMSO-*d*_6_). Infrared spectra were recorded on a Vector 22 spectrometer (Bruker, Bremen, Germany) in Nujol mulls. The main bands are given in cm^−1^. Positive-ion electrospray ionization (ESI) mass spectra were recorded on a double-focusing MAT 95 instrument (Finnigan, Waltham, MA, USA) with BE geometry. Melting points (mp) were determined on a SMP1 Melting Point apparatus (Stuart Scientific, Stone, UK) and are uncorrected. All products reported showed ^1^H NMR spectra in agreement with the assigned structures. The purity of the tested compounds was determined by combustion elemental analyses conducted by the Microanalytical Laboratory of the Chemistry Department of the University of Ferrara with a MT-5 CHN recorder elemental analyzer (Yanagimoto, Kyoto, Japan) and the values found were within 0.4% of theoretical values. Hydrazones **17** and **18** were synthesized as previously described [24].

### 4.2. Synthesis

4.2.1. 1*H*-benzo[*d*]imidazole-2-carbohydrazide (**2**)

A mixture of ethyl 1*H*-benzo[*d*]imidazole-2-carboxylate (**1**, 3.80 g, 20 mmol), and hydrazine monohydrate (3 mL, 61.5 mmol) in EtOH (5 mL) was refluxed for 3 h. After cooling the formed precipitate was filtered off, washed with water (5 × 10 mL) dried and used without further purification. Yield 80%. Mp 240–242 °C (lit. [25] 217–219). IR: 3321, 3265, 3066, 1661, 1609 cm^−1^. ^1^H-NMR: δ 4.62 (s, 2H, NH_2_), 7.28 (m, 2H, Ar), 7.53 (d, *J* = 8.0 Hz, 1H, Ar), 7.70 (d, *J* = 8.0 Hz, 1H, Ar), 10.14 (s, 1H, NH), 13.20 (s, 1H, NH). ESI-MS *m*/*z* 177 (M + H)^+^. Anal. Calcd for C_8_H_8_N_4_O: C, 54.54; H, 4.58; N, 31.80. Found: C, 54.57; H, 4.57; N, 31.76.

#### 4.2.2. General Procedure for the Synthesis of Hydrazones **3**–**19**

A mixture of hydrazide **2** (1 mmol) and the appropriate aldehyde (1 mmol) in EtOH (10 mL) was refluxed for 5 h. After cooling the formed precipitate was filtered off and purified by crystallization from the adequate solvent to give the hydrazone derivatives.

*(E)-N′-(2-Hydroxybenzylidene)-1H-benzo[d]imidazole-2-carbohydrazide* (**3**). Yield 78%. Mp > 250 °C (EtOH). IR: 3191, 1664, 1614, 1556 cm^−1^. ^1^H-NMR: δ 6.94 (m, 2H, Ar), 7.31 (m, 3H, Ar) 7.67 (m, 3H, Ar), 8.83 (s, 1H, CH), 11.23 (s, 1H, OH), 12.78 (s, 1H, NH), 13.51 (s, 1H, NH). ^13^C-NMR: δ 105.4, 109.9, 115.1, 115.9, 123.0, 126.8, 129.7, 136.3, 135.2, 138.4, 141.2, 145.9, 150.1. ESI-MS *m*/*z* 281 (M + H)^+^. Anal. Calcd for C_15_H_12_N_4_O_2_: C, 64.28; H, 4.32; N, 19.99. Found: C, 64.22; H, 4.33; N, 20.05.

*(E)-N′-(3-Hydroxybenzylidene)-1H-benzo[d]imidazole-2-carbohydrazide* (**4**). Yield 78%. Mp > 250 °C (EtOH). IR: 3221, 1677, 1610, 1576 cm^−1^. ^1^H-NMR: δ 6.85 (d, *J* = 8.0 Hz, 1H, Ar), 7.11 (d, *J* = 7.5 Hz, 1H, Ar), 7.21 (s, 1H, Ar), 7.26 (d, *J* = 7.5 Hz, 1H, Ar), 7.29–7.78 (m, 4H, Ar), 8.56 (s, 1H, CH), 9.69 (s, 1H, OH), 12.40 (s, 1H, NH), 13.47 (s, 1H, NH). ^13^C-NMR: δ 105.3, 105,4, 109.8, 117.1, 117.9, 123.1, 130.1, 129.8, 138.7, 138.8, 144.3, 149.9, 150.2. ESI-MS *m*/*z* 281 (M+ H)^+^. Anal. Calcd for C_15_H_12_N_4_O_2_: C, 64.28; H, 4.32; N, 19.99. Found: C, 64.34; H, 4.31; N, 20.03.

*(E)-N′-(4-Hydroxybenzylidene)-1H-benzo[d]imidazole-2-carbohydrazide* (**5**). Yield 80%. Mp > 250 °C (1-PrOH). IR: 3289, 1668,1609, 1584 cm^−1^. ^1^H-NMR: δ 6.86 (d, *J* = 8.5 Hz, 2H, Ar), 7.34 (d, *J* = 7.0 Hz, 2H, Ar), 7.57–7.59 (m, 3H, Ar), 7.77 (d, *J* = 7.0 Hz, 1H, Ar), 8.53 (s, 1H, CH), 10.03 (s, 1H, OH), 12.23 (s, 1H, NH), 13.43 (s, 1H, NH). ^13^C-NMR: δ 105.8, 106,3, 116.8, 118.4, 130.3, 139.8, 144.8, 150.9, 152.3. ESI-MS *m*/*z* 281 (M + H)^+^. Anal. Calcd for C_15_H_12_N_4_O_2_: C, 64.28; H, 4.32; N, 19.99. Found: C, 64.33; H, 4.31; N, 20.04.

*(E)-N′-(2,4-Dihydroxybenzylidene)-1H-benzo[d]imidazole-2-carbohydrazide* (**6**). Yield 75%. Mp > 250 °C (1-PrOH). IR: 3227, 1673, 1638, 1587 cm^−1^. ^1^H-NMR: δ 6.34 (m, 2H, Ar), 6.38 (d, *J* = 6.5 Hz, 1H, Ar), 7.57 (m, 4H, Ar), 8.68 (s, 1H, CH), 10.09 (s, 1H, OH), 11.43 (s, 1H, OH), 12.59 (s, 1H, NH), 13.45 (s, 1H, NH). ^13^C-NMR: δ 105.0, 109.6, 115.1, 115.9, 123.2, 125.9, 127.6, 134.7, 135.1, 137.8, 145.9, 148.0. ESI-MS *m*/*z* 297 (M + H)^+^. Anal. Calcd for C_15_H_12_N_4_O_3_: C, 60.81; H, 4.08; N, 18.91. Found: C, 60.76; H, 4.10; N, 18.94.

*(E)-N′-(2,5-Dihydroxybenzylidene)-1H-benzo[d]imidazole-2-carbohydrazide* (**7**). Yield 81%. Mp > 250 °C (EtOH). IR: 3238, 1681, 1620, 1586 cm^−1^. ^1^H-NMR: δ 6.77 (d, *J* = 9.0 Hz, 1H, Ar), 6.79 (d, *J* = 9.0 Hz, 1H, Ar), 6.96 (s, 1H, Ar), 7.59 (m, 4H, Ar), 8.75 (s, 1H, CH) 9.03 (s, 1H, OH), 10.38 (s, 1H, OH), 12.67 (s, 1H, NH), 13.48 (s, 1H, NH). ^13^C-NMR: δ 105.7, 110.3, 115.6, 116.0, 123.3, 126.5, 130.2, 134.9, 135.6, 138.1, 145.8, 148.1. ESI-MS *m*/*z* 297 (M + H)^+^. Anal. Calcd for C_15_H_12_N_4_O_3_: C, 60.81; H, 4.08; N, 18.91. Found: C, 60.86; H, 4.09; N, 18.87.

*(E)-N′-(2,3,4-Trihydroxybenzylidene)-1H-benzo[d]imidazole-2-carbohydrazide* (**8**). Yield 62%. Mp > 250 °C (EtOH). IR: 3228, 3127, 3061, 1672, 1644 cm^−1^. ^1^H-NMR: δ 6.42 (d, *J* = 8.5 Hz, 1H, Ar), 6.76 (d, *J* = 8.5 Hz, 1H, Ar), 7.64 (m, 4H, Ar), 8.65 (s, 1H, CH), 9.57 (s, 2H, OH), 11.49 (s, 1H, OH), 12.65 (s, 1H, NH), 13.48 (s, 1H, NH). ^13^C-NMR: δ 106.2, 111.0, 115.7, 116.1, 125.6, 127.1, 135.0, 138.6, 138.7, 139.9, 147.1, 149.2. ESI-MS *m*/*z* 313 (M + H)^+^. Anal. Calcd for C_15_H_12_N_4_O_4_: C, 57.69; H, 3.87; N, 17.94. Found: C, 57.64; H, 3.88; N, 17.91.

*(E)-N′-(2,4,6-Trihydroxybenzylidene)-1H-benzo[d]imidazole-2-carbohydrazide* (**9**). Yield 60%. Mp > 250 °C (EtOH). IR: 3225, 1672, 1592 cm^−1^. ^1^H-NMR: δ 5.80 (s, 1H, Ar) 5.86 (s, 1H, Ar), 7.26 (d, *J* = 7.0 Hz, 1H, Ar), 7.30–7.78 (m, 3H, Ar), 8.98 (s, 1H, CH), 10.14 (s, 1H, OH), 11.14 (s, 2H, OH), 12.68 (s, 1H, NH), 13.43 (s, 1H, NH). ^13^C-NMR: δ 99.2, 103.9, 119.7, 127.9, 138.9, 140.1, 145.2, 147.4, 151.0. ESI-MS *m*/*z* 313 (M + H)^+^. Anal. Calcd for C_15_H_12_N_4_O_4_: C, 57.69; H, 3.87; N, 17.94. Found: C, 57.73; H, 3.86; N, 17.92.

*(E)-N′-(2-Hydroxy-4-methoxybenzylidene)-1H-benzo[d]imidazole-2-carbohydrazide* (**10**). Yield 79%. Mp > 250 °C (EtOH). IR: 3214, 1665, 1633, 1607, 1567 cm^−1^. ^1^H-NMR: δ 3.79 (s, 3H, OCH_3_), 6.54 (m, 2H, Ar), 7.48 (m, 5H, Ar), 8.74 (s, 1H, CH), 11.55 (s, 1H, NH), 12.69 (s, 1H, OH), 13.47 (s, 1H, NH). ^13^C-NMR: δ 58.5, 104.4, 109.7, 114.9, 115.8, 123.1, 126.0, 127.6, 134.5, 135.2, 137.7, 145.7, 147.6. δ ESI-MS *m*/*z* 311 (M + H)^+^. Anal. Calcd for C_16_H_14_N_4_O_3_: C, 61.93; H, 4.55; N, 18.06. Found: C, 61.99; H, 4.53; N, 18.02.

*(E)-N′-(3-Ethoxy-2-hydroxybenzylidene)-1H-benzo[d]imidazole-2-carbohydrazide* (**11**). Yield 70%. Mp > 250 °C (EtOH). IR: 3322, 1694, 1610, 1583 cm^−1^. ^1^H-NMR: δ 1.36 (t, *J* = 7.0 Hz, 3H, CH_3_), 4.07 (q, *J* = 7.0 Hz, 2H, CH_2_), 6.86 (m, 1H, Ar), 7.03 (d, *J* = 8.0 Hz, 1H, Ar), (d, *J* = 8.0 Hz, 1H, Ar), 7.59 (m, 4H, Ar), 8.84 (s, 1H, CH), 10.99 (s, 1H, NH), 12.81 (s, 1H, OH), 13.53 (s, 1H, NH). ^13^C-NMR: δ 14.9, 65.0, 106.3, 107.7, 115.6, 115.8, 123.1, 124.5, 129.9, 135.8, 137.9, 146.2, 147.4, 149.2. ESI-MS *m*/*z* 325 (M + H)^+^. Anal. Calcd for C_17_H_16_N_4_O_3_: C, 62.95; H, 4.97; N, 17.27. Found: C, 63.01; H, 4.99; N, 17.23.

*(E)-N′-(3-Hydroxy-4-methoxybenzylidene)-1H-benzo[d]imidazole-2-carbohydrazide* (**12**). Yield 71%. Mp > 250 °C (EtOH). IR: 3277, 3219, 1673, 1612, 1567 cm^−1^. ^1^H-NMR: δ 3.96 (s, 3H, OCH_3_), 6.99 (d, *J* = 8.5 Hz, 1H, Ar), 7.0 (d, *J* = 8.5 Hz, 1H, Ar), 7.29 (s, 1H, Ar), 7.49 (m, 4H, Ar), 8.49 (s, 1H, CH), 9.39 (br s, 1H, OH), 12.25 (br s, 1H, NH), 13.20 (br s, 1H, NH). ^13^C-NMR: δ 58.5, 106.0, 106.3, 110.1, 115.9, 116.7, 124.8, 128.5, 129.7, 135.4, 136.1, 145.4, 146.9. ESI-MS *m*/*z* 311 (M + H)^+^. Anal. Calcd for C_16_H_14_N_4_O_3_: C, 61.93; H, 4.55; N, 18.06. Found: C, 61.88; H, 4.56; N, 18.10.

*(E)-N′-(4-(Diethylamino)-2-hydroxybenzylidene)-1H-benzo[d]imidazole-2-carbohydrazide* (**13**). Yield 78%. Mp > 250 °C (EtOH). IR: 1668, 1631, 1586 cm^−1^. ^1^H-NMR: δ 1.10 (t, *J* = 7.0 Hz, 6H, CH_3_), 3.35 (q, *J* = 7.0 Hz, 4H, CH_2_), 6.12 (s, 1H, Ar), 6.27 (d, *J* = 6.0 Hz, 1H, Ar), 7.14 (d, *J* = 6.0 Hz, 1H, Ar), 7.32 (m, 2H, Ar), 7.57(m, 1H, Ar), 7.77 (m, 1H, Ar), 8.60 (s, 1H, CH), 11.42 (s, 1H, OH), 12.52 (s, 1H, NH), 13.42 (s, 1H, NH). ^13^C-NMR: δ 13.2, 48.9, 99.2, 103.6, 106.7, 109.9, 115.9, 122.7, 125.9, 131.4, 137.7, 140.0, 142.3, 146.2, 150.6. ESI-MS *m*/*z* 352 (M + H)^+^. Anal. Calcd for C_19_H_21_N_5_O_2_: C, 64.94; H, 6.02; N, 19.93. Found: C, 65.01; H, 5.99; N, 19.97.

*(E)-N′-(5-Chloro-2-hydroxybenzylidene)-1H-benzo[d]imidazole-2-carbohydrazide* (**14**). Yield 85%. Mp > 250 °C (EtOH). IR: 3215, 1680, 1605, 1591 cm^−1^. ^1^H-NMR: δ 6.96 (d, *J* = 8.0 Hz, 1H, Ar), 7.33–7.65 (m, 6H, Ar), 8.82 (s, 1H, CH), 11.15 (br s, 1H, OH), 12.90 (br s, 1H, NH), 13.30 (br s, 1H, NH). ^13^C-NMR: δ 105.6, 107.4, 109.9, 115.9, 117.2, 122.8, 123.0, 128.0, 129.5, 134.4, 141.5, 141.9, 147.6, 151.2. ESI-MS *m*/*z* 315 (M + H)^+^. Anal. Calcd for C_15_H_11_ClN_4_O_2_: C, 57.24; H, 3.52; N, 17.80. Found: C, 57.30; H, 3.51; N, 17.83.

*(E)-N′-(5-Bromo-2-hydroxybenzylidene)-1H-benzo[d]imidazole-2-carbohydrazide* (**15**). Yield 82%. Mp > 250 °C (EtOH). IR: 3200, 1680, 1602, 1588 cm^−1^. ^1^H-NMR: δ 6.91 (d, *J* = 9.0 Hz, 1H, Ar), 7.33 (d, *J* = 6.0 Hz, 1H, Ar), 7.35 (d, *J* = 6.0 Hz, 1H, Ar), 7.60 (m, 4H, Ar), 8.81 (s, 1H, CH), 11.15 (s, 1H, OH), 12.85 (s,1H, NH), 13.50 (s, 1H, NH). ^13^C-NMR: δ 105.2, 107.5, 109.9, 116.0, 117.3, 122.9, 123.0, 128.2, 129.4, 134.5, 141.5, 142.0, 147.7, 151.1. ESI-MS *m*/*z* 359 (M + H)^+^. Anal. Calcd for C_15_H_11_BrN_4_O_2_: C, 50.16; H, 3.09; N, 15.60. Found: C, 50.22; H, 3.11; N, 15.57.

*(E)-N′-((2-Hydroxynaphthalen-1-yl)methylene)-1H-benzo[d]imidazole-2-carbohydrazide* (**16**). Yield 85%. Mp > 250 °C (EtOH). IR: 3254, 1679, 1625, 1576 cm^−1^. ^1^H-NMR: δ 7.26 (d, *J* = 9.0 Hz, 1H, Ar), 7.35 (d, *J* = 6.5 Hz, 1H, Ar), 7.38 (d, *J* = 6.5 Hz, 1H, Ar), 7.54 (m, 3H, Ar), 7.82 (d, *J* = 7.0 Hz, 1H, Ar), 8.11 (m, 3H, Ar), 9.79 (s, 1H, CH), 12.77 (s, 1H, NH), 12.86 (s, 1H, OH), 13.58 (s, 1H, NH). ^13^C-NMR: δ 111.6, 115.9, 122.0, 123.2, 123.6, 126.1, 126.8, 127.8, 131.0, 132.1, 135.0, 136.2, 137.8, 145.7, 147.3, 151.8, 158.0, 161.3. MS *m*/*z* 331 (M + H)^+^. Anal. Calcd for C_19_H_14_N_4_O_2_: C, 69.08; H, 4.27; N, 16.96. Found: C, 69.14; H, 4.29; N, 17.01.

*(E)-N′-(Naphthalen-1-ylmethylene)-1H-benzo[d]imidazole-2-carbohydrazide* (**19**). Yield 92%. Mp > 250 °C (EtOH). IR: 3208, 3044, 1618, 1558 cm^−1^. ^1^H-NMR: δ 7.32 (m, 2H, Ar), 7.65 (m, 4H, Ar), 7.81 (m, 1H, Ar), 8.01–8.16 (m, 3H, Ar), 8.74 (m, 1H, Ar), 9.44 (s, 1H, CH), 12.54 (s, 1H, NH), 13.55 (s, 1H, NH). ^13^C-NMR: 115.0, 122.9, 123.1, 126.0, 126.2, 127.5, 128.4, 128.5, 130.2, 130.6, 130.7, 138.6, 143.3, 144.8, 155.6. ESI-MS *m*/*z* 315 (M + H)^+^. Anal. Calcd for C_19_H_14_N_4_O: C, 72.60; H, 4.49; N, 17.82. Found: C, 72.66; H, 4.47; N, 17.78.

### 4.3. Antiproliferative Activity

#### 4.3.1. Cell Lines

Human cervical carcinoma (HeLa), human T-lymphoblast (CEM) and mouse leukema (L1210) cells were obtained from ATCC (Middlesex, UK). Human pancreatic carcinoma (Mia-Paca 2) cells were kindly provided by Prof. Anna Karlsson (Karolinska Institute, Stockholm, Sweden). All cell lines were grown in Dulbecco’s modified Eagle’s medium (DMEM; Gibco, Carlsbad, CA, USA), supplemented with 10% fetal bovine serum (FBS, Gibco), 0.01M Hepes (Gibco) and 1 mM sodium pyruvate (Gibco) in a humidified 5% CO_2_ incubator at 37 °C.

#### 4.3.2. Cell Proliferation

Suspension (L1210 and CEM cells) were seeded in 96-well microtiter plates at 60,000 cells/well in the presence of different concentrations of the compounds. The cells were allowed to proliferate for 48 h or 96 h, respectively and then counted in a Coulter counter. The 50% inhibitory concentration (IC_50_) was defined as the compound concentration required to reduce cell proliferation by 50%. HeLa and Mia-Paca2 cells were seeded in 96-well plates at 15,000 cells/well in the presence of different concentrations of the compounds. After 4 days of incubation, the cells were trypsinized and counted in a Coulter counter.

#### 4.3.3. IC_50_ Determination

The compounds were dissolved in DMSO at 20 mM (stock solution) and kept in the refrigerator until use. Then, compound dilutions were made in cell culture medium, and serial compound concentrations were tested starting at 100 μM as the highest concentration. The DMSO concentration, present in the highest compound concentration was 0.5% that is a concentration that did not affect the tumor cell proliferation. The IC_50_ values were calculated using following formula: C1 − [ 50 − N1%/N2% − N1% ] × ( C1 − C2) wherein C1 is the compound concentration that inhibits cell proliferation more than 50%; C2 is the compound concentration that inhibits cell proliferation less than 50%; N1% represents the cell number (in percent of control in the absence of compound) obtained in the presence of C1 and N2% represents the cell number (in percent of control in the absence of compound) obtained in the presence of C2.

## 5. Conclusions

This study started with the aim to explore the possible antiproliferative properties of dualistic molecules bearing a combination of the hydrazone and benzimidazole moieties. Based on compounds **3**–**19** the *in vitro* activity on murine leukemia (L1210), human T-lymphoblastic leukemia (CEM), human cervix carcinoma (HeLa) and human pancreas carcinoma (Mia Paca-2) cells, we have observed that the presence of a 2-hydroxyl group on the arylidene moiety favourably modulates antiproliferative activity. In particular, hydrazones 10 and 16 inhibited the growth of all tested cell lines with low (<10 µM) micromolar IC_50_ values. Predictions of the ADME properties for studied compounds showed that all hydrazones **3**, **6**–**8**, **10**, **11**, **13**–**16** might present good passive oral absorption. In conclusion by combination of benzimidazole and hydrazone pharmacophores we obtained a new class of *N*′-(4-arylidene)-1*H*-benzo[*d*]imidazole-2-carbohydrazides endowed with significant antiproliferative activity as well as good potential absorption properties. These results further encourage us in developing an enlarged synthetic study in order to highlight SAR in this interesting class of molecules.

## Figures and Tables

**Table 1:** Antiproliferative activity of *N*′-(4-arylidene)-1*H*-benzo[*d*]imidazole-2-carbohydrazides **3**–**19**.

Compd.	Ar		IC_50_ (μM) ^a^		
		**L1210**	**CEM**	**HeLa**	**Mia-Paca-2**
**3**	2-OH-phenyl	5.7 ± 0.9	4.4 ± 0.2	12 ± 0.7	34 ± 0.9
**4**	3-OH-phenyl	94 ± 7	98 ± 10	88 ± 12.5	>100
**5**	4-OH-phenyl	47 ± 2	85 ± 0.01	>100	>100
**6**	2,4-(OH)2-phenyl	2.6 ± 0.9	2.6 ± 1.0	4.7 ± 1.6	21 ± 12
**7**	2,5-(OH)2-phenyl	22 ± 7	4.9 ± 1.2	77 ± 18	>100
**8**	2,3,4-(OH)3-phenyl	47 ± 7	20 ± 8	97 ± 28	>100
**9**	2,4,6-(OH)3-phenyl	90 ± 7	>100	>100	>100
**10**	2-OH-4-OMe-phenyl	1.6 ± 0.9	0.98 ± 0.02	4.0 ± 0.4	6.3 ± 3.2
**11**	2-OH-3-OEt-phenyl	5.9 ± 2.4	6.3 ± 1.9	22 ± 0.02	23 ± 8
**12**	3-OH-4-OMe-phenyl	92 ± 4	57 ± 3.7	>100	>100
**13**	2-OH-4-N(Et)2-phenyl	14 ± 2	4.8 ± 0.8	23 ± 3	40 ± 6
**14**	2-OH-5-Cl-phenyl	7.4 ± 2.5	1.8 ± 0.6	4.8 ± 0.9	9.2 ± 4.0
**15**	2-OH-5-Br-phenyl	5.0 ± 2.8	1.8 ± 0.9	4.9 ± 0.4	35 ± 5
**16**	2-OH-naphtyl	2.9 ± 1.3	1.0 ± 0.01	2.5 ± 1.4	7.9 ± 0.3
**17**	phenyl	>250	>250	>250	220 ± 37
**18**	4-OMe-phenyl	>250	>250	>250	>250
**19**	napht-1-yl	240±13	>250	>250	>250

**^a^** IC_50_ values (compound concentration that reduces cell growth by 50%) are shown.

**Table 2:** In silico physico-chemical properties (ADME) of active compounds **3**, **6**–**8**, **10**, **11**, **13**–**16**.

Compd.	TPSA	n-ROTB	MV	MW	miLogP	n-ON	n-OHNH	n-Viol
**3**	90.37	3	242.94	280.29	2.64	6	3	0
**6**	110.60	3	250.96	296.29	2.13	7	4	0
**7**	110.60	3	250.96	296.29	2.13	7	4	0
**8**	130.83	3	258.97	312.29	1.67	8	5	0
**10**	99.61	4	268.48	310.31	2.677	7	3	0
**11**	99.61	5	285.29	324.34	2.62	7	3	0
**13**	93.61	6	322.45	351.41	3.47	7	3	0
**14**	90.37	3	256.47	314.73	3.29	6	3	0
**15**	90.37	3	260.82	359.18	3.42	6	3	0
**16**	90.37	3	286.93	330.35	3.79	6	3	0

TPSA: topological polar surface area; n-ROTB: number of rotable bonds; MV: molecular volume; MW: molecular weight; milogP: logarithm of partition coefficient between *n*-octanol and water; n-ON: number of hydrogen bond acceptors; n-OHNH: number of hydrogen bond donors; n-Viol: number of violations of Lipinski’s rule.

## Abbreviations

The following abbreviations are used in this manuscript:

ADME	Adsorption, distribution, metabolism and elimination
IR	infrared spectra
NMR	Nuclear magnetic resonance
MS	Mass spectra
TPSA	Total polar surface area
